# The association between screen use and central obesity among children and adolescents: a systematic review and meta-analysis

**DOI:** 10.1186/s41043-023-00391-5

**Published:** 2023-06-02

**Authors:** Mohammad Ghasemirad, Leyla Ketabi, Ehsan Fayyazishishavan, Ali Hojati, Zahra Hosseinzadeh Maleki, Mohammad Hadi Gerami, Mahdi Moradzadeh, Jaime Humberto Ortiz Fernandez, Reza Akhavan-Sigari

**Affiliations:** 1 Department of Periodontics, Faculty of Dentistry, Rafsanjan University of Medical Sciences, Rafsanjan, Iran; 2 Department of Pediatrics, School of Medicine, Bouali Hospital, Ardabil University of Medical Sciences, Ardabil, Iran; 3 Department of Biostatistics and Data Science, School of Public Health, The University of Texas and Health Science Center at Houston (UTHealth), Houston, USA; 4 Student Research Committee, Tabriz University of Medical Sciences, Tabriz, Iran; 5 Department of Psychology, Faculty of Education Sciences and Psychology, Ferdowsi University of Mashhad, Mashhad, Iran; 6 Bone and Joint Disease Research Center, Shiraz University of Medical Sciences, Shiraz, Iran; 7 Department of Medical Sciences, Tabriz Branch of Islamic Azad University, Tabriz, Iran; 8 Facultad de Ingeniería, Universidad Peruana Los Andes, Huancayo, Peru; 9 Department of Neurosurgery, University Medical Center Tuebingen, Tuebingen, Germany; 10 Department of Health Care Management and Clinical Research, Collegium Humanum Warsaw Management University, Warsaw, Poland

**Keywords:** Central obesity, Abdominal obesity, Screen time, Television, Video games, Personal computer

## Abstract

Currently, there is no summative study evaluating the association between central obesity and screen time. This systematic review and meta-analysis aimed to summarize the results of studies evaluating the association between screen time and central obesity among children and adolescents. To this end, we performed a systematic search in three electronic databases, including Scopus, PubMed, and Embase to retrieve the related studies up to March 2021. Nine studies were eligible to be included in the meta-analysis. There was no association between odds of central obesity and screen time [odds ratio (OR) 1.136; 95% CI 0.965–1.337; *P* = 0.125]; however, waist circumference (WC) was 1.23 cm higher in those with highest screen time versus those in the lowest screen time category [weighted mean difference (WMD) = 1.23; 95% 95% CI 0.342–2.112; *P* = 0.007; Fig. 3]. Moreover, the possible sources of heterogeneity in the included studies were continent and sample size. No evidence of publication bias was reported. For the first time, the current systematic review and meta-analysis revealed that those with the highest screen time had higher WC compared with those with the lowest screen time. Although, there was no association between odds of central obesity and screen time. Due to the observational design of the included studies, it is impossible to infer the cause-effect relationship. Therefore, further interventional and longitudinal studies are warranted to better elucidate the causality of these associations.

**Supplementary Information:**

The online version contains supplementary material available at 10.1186/s41043-023-00391-5.

## Introduction

In recent years, technological developments and socio-demographic progress have been introduced as the inevitable reasons for the growing prevalence of obesity due to a sedentary lifestyle and the so-called “obesogenic environment” [1–3]. The World Health Organization (WHO) recommends children and adolescents to have at least one hour of moderate to vigorous physical activity per day and vigorous-intensity physical activity at least three times per week [4]. Nonetheless, nowadays, the time spent actively is increasingly being replaced by screen-based media, and screen time has increased in both children and adolescents with a pronounced role in obesity promotion worldwide [5–8].

Among obesity measurements, central obesity or abdominal obesity is an important prognostic factor of metabolic disorders, including hypertension, insulin resistance, fatty liver, and diabetes among children and adolescents [9, 10]. Moreover, people with higher body adiposity are more susceptible to cardio-metabolic risk factors. According to the results of the Bogalusa Heart Study, abdominal fat distribution, determined by high waist circumference (WC) among 5–17-year-old children and adolescents, was associated with abnormal levels of serum lipids [11–13]. In a systematic review of more than 3966 articles, high WC among children and adolescents was associated with high blood pressure and dyslipidemia [14]. A sedentary life style is associated with central obesity; according to the findings of a community-based study among 124,113 children (9.9 ± 1.1 years old, 51% boys), sedentary activities were associated with increased odds of central obesity [odds ratio (OR) 1.10, 95% CI 1.07–1.14] [15].

Reduced physical activity and increased sedentary behaviors are serious health problems among children and adolescents [16, 17]. Some recent studies reported that 10–12-year-old children had 8 h of sedentary behaviors per day and they spent more than 2 h per day in front of computer or television (TV) screens [18–20]. One of the important sedentary behaviors among children is screen time, which collectively refers to the time spent on watching television, playing video games, and working with a computer [21]. Screen time is dramatically increased as a result of increased use of technology, such as electronic media, TV, video, computer, tablet, and internet games, or the use of cell phones full of built-in games [22]. The American Academy of Pediatrics (AAP) recommends limiting screen time among children and adolescents to less than 2 h per day with no screens for kids under two years old, and less than an hour per day for kids aged 2–5 years [23, 24].

Although numerous studies have investigated the relationship between screen time and general obesity, limited studies have evaluated the association between screen time and central obesity and reported inconsistent results. In a cross-sectional study including 930 adolescents, Castro JAC et al*.* [9] evaluated the association between TV, personal computer (PC), and video games with central obesity (defined as having WC ≥ 85th percentile for age and sex), and reported that TV time more than 2 h per day was associated with increased odds of central obesity (OR 2.11; 95% CI 1.08–4.13; *P* < 0.01). However, no association was found between using PC and playing video games with central obesity. In another study by Berentzen NE et al. [25], those with the highest WC had significantly higher screen time (*P* = 0.0001). However, several other studies did not report a positive association between screen time and central obesity, or they reported a non-significant association. For example, Kerkadi A et al*.* [26, 27] reported a non-significant increase in the odds of central obesity in individuals with screen time higher than 2 h per day compared to those with screen time lower than 2 h per day.

In this study, we assumed that high screen time among children and adolescents might be associated with increased odds of central obesity. Accordingly, this systematic review and meta-analysis and we have two study questions: (1) what is the association between screen time and central obesity among children and adolescents? and (2) how do the different parameters like screen device type, screen time measurement tool, and the demographic characteristics of studies’ participants including participants’ age, geographical distribution, and setting of the studies affect the association between screen time and central obesity among children and adolescents?

## Materials and methods

To report the results, we used the Preferred Reporting Items for Systematic Reviews and Meta‐Analyses (PRISMA) (Additional file 1: Table S1) [28]. The project’s registration code in PROSPERO was CRD42021233899.

### Search strategy and selection of studies

**Fig. 1:**
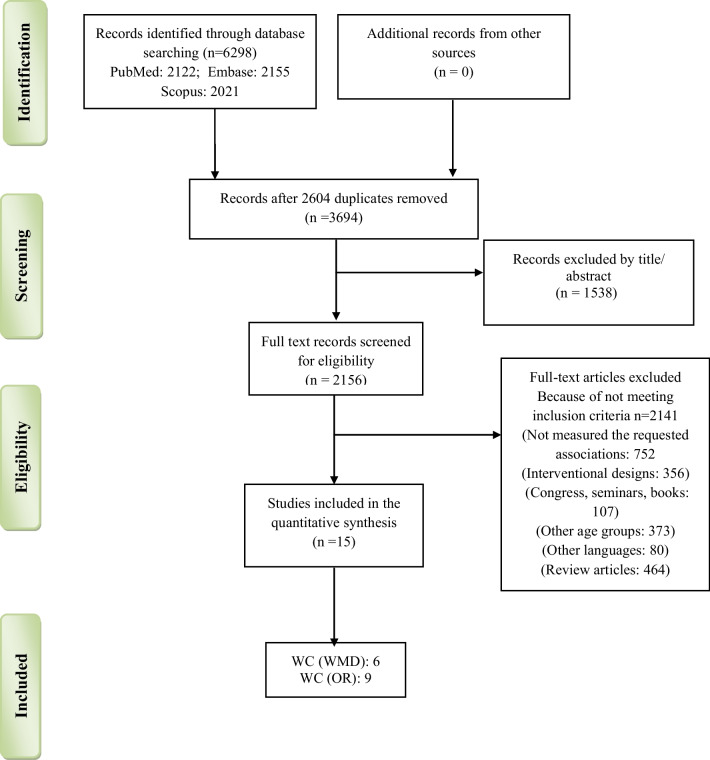
Study flowchart

An updated systematic search was performed in three electronic databases, including PubMed, Scopus, and Embase up to March 2021, and a total of 6298 articles were obtained (Fig. 1). The search strategy used a combination of the MeSH (Medical Subject Headings) terms from the PubMed database and free text words (Additional file 1: Table S2). To avoid missing the studies that measured central obesity as a secondary or tertiary measurement variable, we also included some keywords related to general obesity like body mass index (BMI), body fatness, obesity, and overweight. After removing the duplicate studies, we screened the titles and abstracts of the remainig studies. As a result, 2156 articles remained for further analysis. Also, two independent researchers checked the references independently. Then, we removed 2141 manuscripts due to irrelevant subject, irrelevant design, involving other age groups, published in other languages, and not evaluating the association between the study parameters. Meanwhile, we excluded all review, conference, and seminar papers. Any discrepancies between reviewers were resolved by discussion.

### Inclusion and exclusion criteria

The following inclusion criteria were applied in our research: (1) studies with an observational design (case–control, cross-sectional, or cohort studies with the baseline measurement of study parameters); (2) studies evaluating the relationship between screen time and central obesity (3); studies including children and adolescents (≤ 18 years); (4) studies providing the mean ± standard deviation (SD) of WC or waist to hip ratio (WHR) among highest versus lowest screen users or those reporting the odds of central obesity in the highest versus lowest screen users.

All clinical trials, systematic reviews, meta-analyses, case reports, case series, experimental studies, short communications, and letters to editors were excluded. Moreover, we excluded the studies published in languages other than English and studies not examining the relationship between screen time and obesity.

### Data extraction and quality assessment

We extracted the following data from the included studies: first author’s name, journal name, year of publication, participants’ age range, gender, and health status, study design, sample size, setting, definition of central obesity and screen time, screen time measurement tool, and the main results. The methodological quality of the included studies was assessed using the Agency for Healthcare Research and Quality (AHRQ) checklist [29] (Table 1).

**Table 1:** Agency for Healthcare Research and Quality (AHRQ) checklist to assess the quality of the cross-sectional studies

ARHQ Methodology Checklist items for Cross-Sectional study	Castro AC [9]	Berentzen NE [71]	Zhang Y [39]	De-Lima TR [38]	Kerkadi A [26]	Huang HM [34]	Byun W [33]	Safiri S [41]	Suchert, V [40]
(1) Define the source of information (survey, record review)	⊕	⊕	⊕	⊕	⊕	⊕	⊕	⊕	⊕
(2) List inclusion and exclusion criteria for exposed and unexposed subjects (cases and controls) or refer to previous publications	⊕	⊕	⊕	⊕	⊕	⊕	⊕	⊕	⊕
(3) Indicate time period used for identifying patients	–	⊕	⊕	⊕	_	⊕	⊕	_	⊕
(4) Indicate whether or not subjects were consecutive if not population-based	⊕	–	⊕	–	⊕	–	⊕	⊕	–
(5) Indicate if evaluators of subjective components of study were masked to other aspects of the status of the participants	–	–	–	–	U	–	–	U	–
(6) Describe any assessments undertaken for quality assurance purposes (e.g., test/retest of primary outcome measurements)	–	–	–	–	U	–	–	U	⊕
(7) Explain any patient exclusions from analysis	⊕	⊕	⊕	⊕	–	⊕	⊕	–	⊕
(8) Describe how confounding was assessed and/or controlled	⊕	⊕	⊕	⊕	⊕	–	⊕	⊕	⊕
(9) If applicable, explain how missing data were handled in the analysis	⊕	⊕	⊕	–	⊕	–	⊕	⊕	⊕
(10) Summarize patient response rates and completeness of data collection	–	⊕	⊕	⊕	⊕	⊕	⊕	⊕	⊕
(11) Clarify what follow-up, if any, was expected and the percentage of patients for which incomplete data or follow-up was obtained	–	–	–	–	⊕	–	–	⊕	–
	6	7	8	6	7	5	8	7	8

### Definitions

According to the definition by the Oxford English Dictionary, screen time is “the time spent using a device such as a computer or games console” [30]. In the current meta-analysis, screen time was defined as “time spent passively watching screen-based entertainment (TV, computer, mobile devices). As defined by the WHO, this does not include active screen-based games where the physical activity or movement is required” [31].

In the current meta-analysis, a child was defined as an age under 10 years old and an adolescent as an age of 10–19 years old, as previously described by the WHO [32].

Central obesity was defined by the international age and sex specific cut-offs of WC [≥ 85th percentile and more than 90th percentile] and the thresholds of WHR [WHR ≥ 0.5] [9, 33, 34].

### Statistical analysis

STATA software version 13 (STATA Corp, College Station, TX, USA) was used for data analysis, and *P*-values less than 0.05 were considered statistically significant. We included the studies that reported the comparison between WC or WHR [mean (SD)] in the highest versus lowest screen time category and the studies that evaluated the odds of central obesity in the meta-analysis. Therefore, the weighted mean difference (WMD) or the odds ratio (OR) with a 95% confidence interval (CI) was reported. The method of Hozo et al*.*[35] was used when the median and range were reported instead of mean and SD, and the median values were considered as best estimates of mean when the sample size of the study was more than 25 and the SD was calculated as follows: $S^{2} \approx \left( {\frac{1}{12}(\frac{{\left( {a - 2m + b} \right)^{2} }}{4} + \left( {b - a} \right)^{2} } \right)$. Also, Walter and Yao’s method was used for calculating the missing SDs, as follows: SD = (b − a)/4 [36, 37]. If the number of participants in categories was not provided, an equal number of participants in each category was assumed. Cochran's Q and I-squared tests were used to identify between-study heterogeneity. The possible sources of heterogeneity were identified using subgrouping and meta-regression analysis. Begg’s Funnel plots followed by Begg's adjusted rank correlation and Egger's regression asymmetry tests were used to assess the publication bias.

## Results

### Study characteristics

Nine individual studies were included in the two-class meta-analysis of the association between increased screen time and odds of central obesity [9, 26, 33, 34, 38, 39] (Table 2). Since the study by Castro JAC et al*.* [9] reported the results related to three screen devices (TV, PC, and VF) separately, they were included as three independent studies. Similarly, the study by Huang HM et al*.* [34] reported separate results for TV and PC, and the study by Byun et al*.* [33] reported separate results for TV and PC/video games. Hence, these studies were included as two independent studies.

**Table 2:** The characteristics of the studies included in the two-class meta-analysis regarding the odds of central obesity in children and adolescents with high screen time versus low screen time

Journal / Year	First author	Country	Setting/ num	Age (y)	Central obesity definition	Screen tools	High screen time definition	Adjusted covariates	Main findings
BMC Public Health/ 2020	Zhang Y [39]	China	School/ 2264	12–15	WC ≥ 90th percentile	TV, PC, VG	≥ 2 h /d	Age, sex, being the single child, ethnic minority, fruit and vegetable intake, sleep time, parents’ education, and fathers’ occupation	Non-significant increase in odds of central obesity in people with more than 2 h of screen time compared to those with less than 2 h of screen time (OR 1.31; 95% CI = 0.81–2.11)
Revista Paulista de Pediatria/ 2020	De Lima TR[38]	Brazil	School/ 583	11–17	WC ≥ 90th percentile	TV, PC, VG	≥ 4 h /d	Gender, maternal schooling, alcohol consumption, cigarette smoking	No significant association between odds of central obesity and screen time
Int J Env Res Public Health/ 2019	Kerkadi A [26]	Qatar	Community/ 1161	14–18	WHR > 0.5 and WC ≥ 90th percentile	TV, PC, VG	≥ 2 h /d	Age, nationality	Non- significant increase in odds of central obesity in higher than 2 h ST versus lower than 2 h (OR 1.11; 95% CI = 0.83–1.42)
Revista Paulista de Pediatria/ 2016*	Castro JAC [9]	Brazil	School/ 930	14–19	WC ≥ 85th percentile	TV, PC, VG	≥ 2 h /d	Gender, skin color, and age in the distal block, economic level, maternal education, and school shift in the intermediate block, and physical activity, alcohol consumption, soft drink consumption, sleep, and sedentary behavior in distal block	Significantly higher odds of central obesity in those with higher than 2 h/d TV watching time versus those with the lower than 2 h/d TV watching time (OR 2.11; 95% CI 1.08–4.13; *P* = 0.02). This association was not significant for PC (OR 0.66; 95% CI 0.40–1.07; *P* = 0.09) or VG (OR 0.66; 95% CI 0.40–1.07; *P* = 0.78)
J Nurs Res/ 2013	Huang HM [34]	Taiwan	Community/ 275	9–10	WHR > 0.5	TV, PC games, internet	≥ 2 h /d		Significantly higher odds of central obesity in people watching TV for more than 2 h compared to those with less than 2 h (OR 1.64; 95% CI 1.09–2.48; *P* = 0.016). This association for PC was non-significant (OR 1.56; 95% CI 0.87–2.67; *P* = 0.130)
Pediatrics/ 2012	Byun W [33]	Korea	Community/ 577	12–18	WC ≥ 85th percentile	PC, Video games	≥ 1 h /d	Age, sex, annual household income, and moderate-to-vigorous physical activity	Higher odds of central obesity in those with high daily TV watching (OR 1.27; 95% CI = 1.06–1.51) and high daily PC use and playing video games (OR 1.20; 95% CI = 1.03–1.40)

*TV* television, *PC* personal computer, *VG* video game, *OR* odds ratio, *WC* waist circumference, *WHR* waist to hip ratio, *CI* confidence interval, *ST* screen time

^*^We included the results of the study by Castro JAC et al*.* [9] as three separate studies (TV, PC, VG). We also considered the study by Huang HM et al*.* [34], that reported separate results for TV and PC, as two separate studies. Also, the study by Byun et al*.* [33], that reported separate results for TV and PC/video games, was included as two separate studies. All of the studies were performed in both genders, used questionnaire for screen time measurement, had cross-sectional design, and the participants were apparently healthy

The meta-analysis included 8484 individuals. One study reported significantly higher odds of central obesity in children and adolescents with a screen time higher than 2 h per day compared to those with a screen time lower than 2 h per day (OR 1.67; 95% CI 1.17–2.61; *P* = 0.005). Other studies reported non-significant higher odds of central obesity in children and adolescents in the high screen time category [9, 26, 34, 38, 39].

Regarding the comparison of WC between highest versus lowest screen time categories, six individual studies with a total number of 10,791 participants were included (Table 3). In the study by Castro JAC et al.[9], the results of three screen devices (i.e., TV, PC, and VG) were reported as three independent studies. According to their results, WC was higher only among those playing video games for more than 2 h per day (*P* = 0.01); for other types of screens, no significant difference was observed. Berentzen et al*.* [25] conducted a community-based study among 1447 children and adolescents aged 10–14 years and reported a significantly higher WC in the highest versus lowest quintile of centrally obese children and adolescents (*P* = 0.0001). The age range of participants was 9–19 years old. Screen time and screen time-based sedentary behavior was investigated using validated questionnaires for children and adolescents of that particular region. The screen time was reported separately for TV, PC, and video games on weekdays and weekends; then the results were summed up to report the total screen time. All the mentioned studies involved a combination of male and female healthy children and adolescents. In addition, two studies were recruited in Brazil [9, 38], one in China [39], one in Qatar [26], one in Taiwan [34], one in Germany [40], one in the Netherlands [25], one in Iran [41], and one in South Korea [33].

**Table 3:** The characteristics of studies included in the two-class meta-analysis regarding the comparison of WC in children and adolescents with high screen time versus low screen time

Journal / Year	First author	Country	Setting/ num	Age (y)	Central obesity definition	Disease status	Screen tools	High screen time definition	Main findings
Revista Paulista de Pediatria/ 2016*	Castro JAC [9]	Brazil	School/ 930	14–19	WC ≥ 85th percentile	Healthy	TV, PC, VG	≥ 2 h /d	WC was significantly higher among those with video game time more than 2 h (*P* = 0.01). For other screens, the difference was not significant (for PC, *P* = 0.83; for TV, *P* = 0.23)
Int J Obes (Lond)/ 2014	Berentzen NE [25]	Netherland	Community/ 1447	11	WC ≥ 85th percentile	Healthy	TV, PC	≥ 2 h /d	Significantly higher screen time in the highest versus lowest quintile of central obesity (*P* = 0.0001)
J Adolesc/ 2016	Suchert, V [40]	Germany	School/ 1228	12–17	WC ≥ 85th percentile	Healthy	TV, DVD, PC	≥ 2 h /d	Significantly higher WC in those with more than 2 h/day screen time compared with those with less than 2 h per day (*P* = 0.003)
Iran J Public Health/ 2015	Safari S [41]	Iran	School/ 5326	10–18	WC ≥ 85th percentile	Healthy	TV, PC	≥ 2 h /d	Those with the highest screen time had significantly higher WC (*P* < 0.05)

*TV* television, *PC* personal computer, *VG* video game, *OR* odds ratio, *WC* waist circumference, *WHR* waist to hip ratio, *CI* confidence interval, *ST* screen time

^*^We included the results of the study by Castro JAC et al*.* [9] as three separate studies (TV, PC, VG). All the studies were performed in both genders, used questionnaire for screen time measurement, had cross-sectional design, and the participants were apparently healthy

### Results of the meta-analysis

**Fig. 2:**
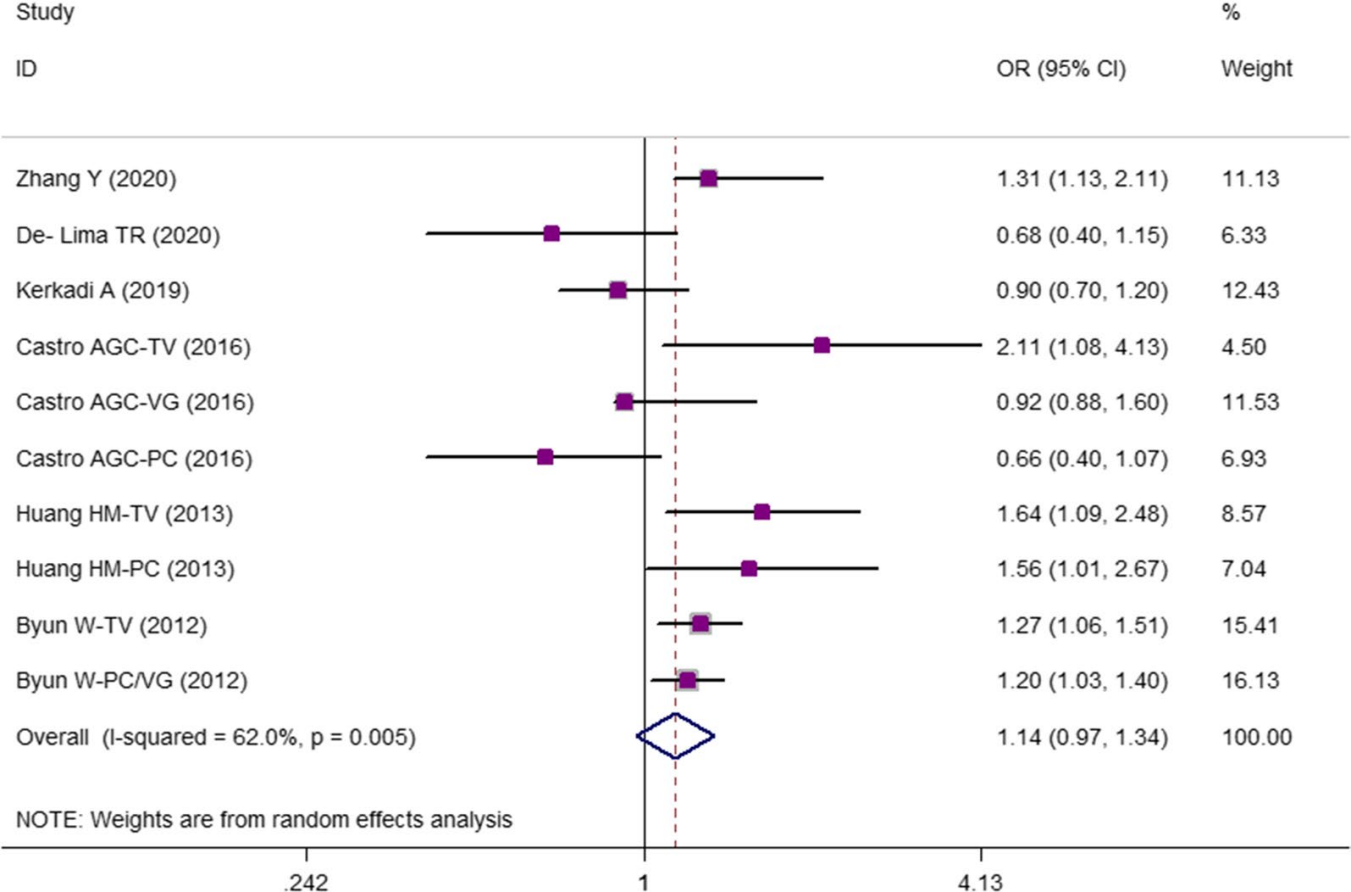
The association between screen time and pooled odds ratio (OR) of central obesity in highest versus lowest screen users. I2 represents the degree of heterogeneity

**Fig. 3:**
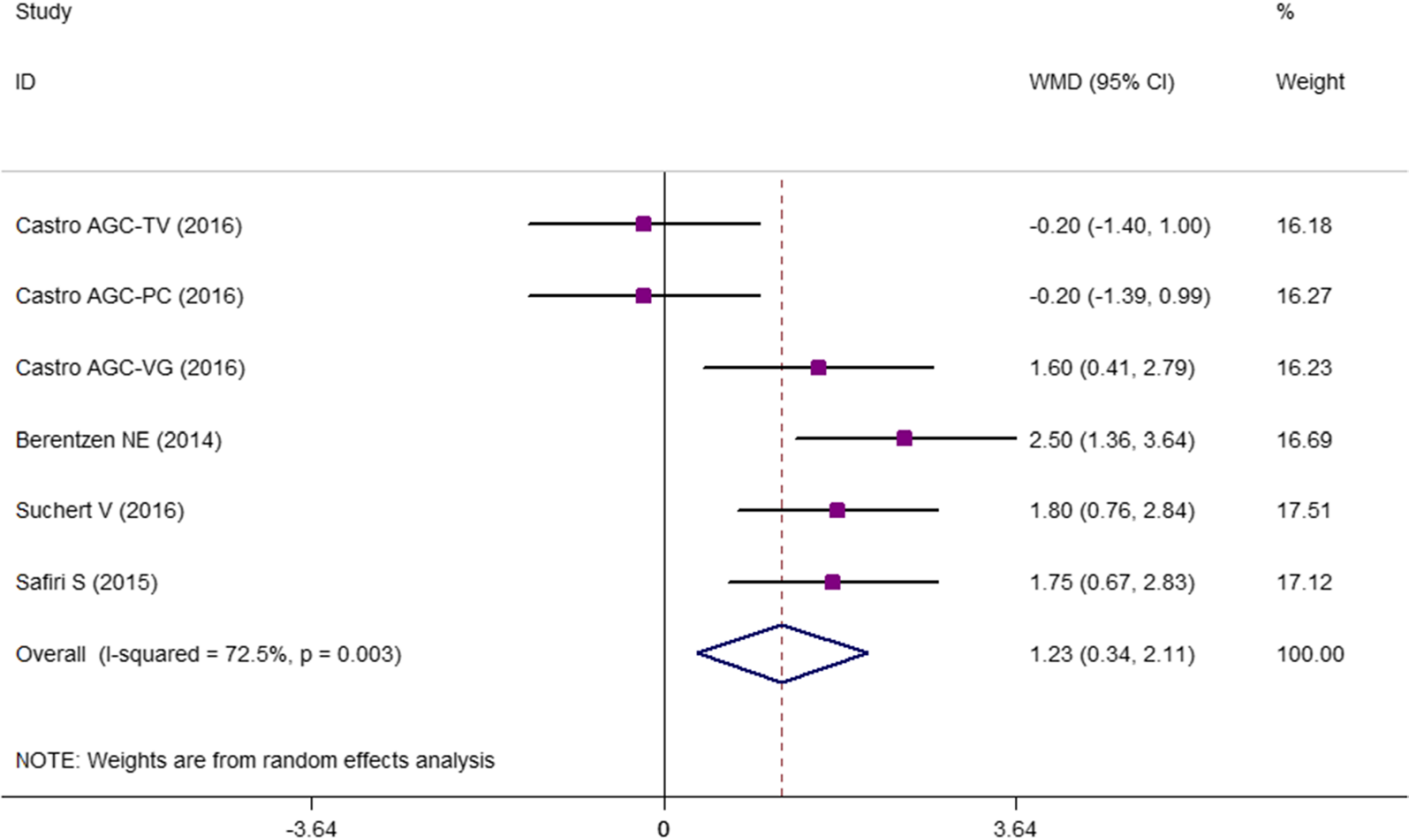
Weighted mean difference (WMD) with 95% confidence interval (CI) of WC in highest versus lowest screen users. I2 represents the degree of heterogeneity

According to the results of the current meta-analysis, there was no association between odds of central obesity and screen time in children and adolescents (OR 1.136; 95% CI 0.965–1.337; *P* = 0.125) (Fig. 2). Since between-study heterogeneity was higher than 50%, we performed subgrouping. The results showed that continent and age range were the possible sources of the observed between-study heterogeneity (Table 4). Also, in a sensitivity analysis using the leave-one-out method, by removing the video game result of the study by Castro JAC et al*.* [9], the results were significant (OR 1.196; 95% CI 1.024–1.397; *P* = 0.024). Moreover, WC was 1.23 cm higher in children and adolescents with the highest screen time compared to those with the lowest screen time (WMD = 1.23; 95% CI = 0.342–2.112; *P* = 0.007; Fig. 3). The results of subgrouping indicated no heterogeneity for studies with a sample size of more than 1000 people (Table 5). In addition, no evidence of publication bias was reported according to the visual asymmetry of funnel plots and the results of Begg’s and Egger’s tests [95% OR of central obesity and screen time: Egger’s test (*P* = 0.162) and Begg’s test (*P* = 0.677); WC in the highest versus lowest screen time category: Egger’s test (*P* = 0. 0.213) and Begg’s test (*P* = 0.188) (Additional file 1: Fig. S1)].

**Table 4:** Subgroup analysis for the odds of central obesity in children and adolescents with high screen time versus those with low screen time

Group	No. of studies*	OR (95% CI)			P_within group_	P_between group*_	P_heterogeneity_	I^2^, %
Total	9	1.136	0.965	1.337	0.125		0.036	62.0
Continent						< 0.001		
America	3	1.065	0.571	1.986	< 0.001		0.032	71.0
*Asia*	6	1.225	1.069	1.404	< 0.001		0.153	38.0
Screen type						< 0.001		
TV	3	1.447	1.122	1.867	0.004		0.221	33.7
PC	1	1.560	0.890	2.733	0.120		–	–
VG	1	0.920	0.529	1.598	0.767		–	–
TV + VG + PC	4	1.054	0.840	1.321	0.651		0.053	67
Age group						< 0.001		
Adolescents	7	1.135	0.840	1.321	0.132		0.046	53.2
Both	2	1.612	1.157	2.245	0.005		0.888	0.0
Setting						< 0.001		
School	4	1.129	0.747	1.705	0.565		0.041	63.6
Community	5	1.216	1.035	1.430	0.018		0.098	48.9
Sample size						< 0.001		
1000 >	5	1.263	0.851	1.875	0.246		0.028	63.3
≥ 1000	4	1.173	1.022	1.347	0.023		0.171	40.2

*All the included studies had moderate quality. So, no subgrouping was performed

**Table 5:** Subgroup analysis for the comparison of WC in children and adolescents with high screen time versus those with low screen time

Group	No. of studies*	WMD (95% CI)			P_within group_	P_between group*_	P_heterogeneity_	I^2^, %
Total	6	1.227	0.342	2.112	0.007		0.003	72.5
Continent						< 0.001		
America	3	0.400	− 0.776	1.577	0.505		0.055	65.6
Europe	2	2.117	1.352	2.883		< 0.001	0.372	0.0
Iran	1	1.750	0.666	2.834	0.002		–	–
Screen type						< 0.001		
TV	1	− 0.200	− 1.401	1.001	0.744		–	–
PC	1	− 0.200	− 1.389	0.989	0.742		–	–
VG	1	1.600	0.406	2.794	0.009		–	–
TV + PC	1	2.500	1.363	3.637	< 0.001		–	–
TV + PC + VG	2	1.776	1.027	2.525	< 0.001		0.948	0.0
Setting						< 0.001		
Community	1	2.500	1.363	3.637	0.036		–	–
School	5	0.975	0.063	1.886	< 0.001		0.012	68.8
Sample size						< 0.001		
1000 >	3	0.400	− 0.776	1.577	0.505		0.055	65.6
≥ 1000	3	1.995	1.370	2.621	< 0.001		0.585	0.0

*All of the included studies had moderate quality and were performed among adolescents, therefore, subgrouping according to these variables was performed

## Discussion

In this meta-analysis, we provided the quantified results of the association between increased screen time and odds of central obesity in children and adolescents. Considering the results of more than 12,563 children and adolescents, we found no significant association between odds of central obesity and screen time. However, WC was significantly higher (1.2 cm) in those with the highest screen time compared to those with the lowest screen time. Most of the included studies were performed on adolescents and only two studies were performed among both children and adolescents.

One important reason for performing the current meta-analysis was the discrepancy between the results of different studies. For example, in the study by Castro JA et al*.* [9], only those with TV time of more than 2 h per day had greater odds of abdominal obesity while higher PC use or video games were associated with a non-significantly lower chance of abdominal obesity. In contrast, several other studies revealed higher odds of central obesity by increased time of using PCs or playing video games [33, 34, 42]. Therefore, it was necessary to perform a summative study to obtain more accurate results.

The role of several confounders like age, gender, and physical activity level should also be mentioned. In the current meta-analysis, all the included studies were performed in both genders; so, it was impossible to infer a gender-specific result. Although females seemed to have higher body fatness [43], some evidence showed that the prevalence of abdominal obesity was increasing in males [44, 45]; therefore, there is no consensus about the association between central obesity and gender [46]. In another study, boys had a higher screen time compared to girls; at the same time, boys also had higher physical activity levels [47].

Although most of the included studies were adjusted for confounders, including age, gender, diet, and physical activity, there was still an association between obesity and screen time after this adjustment [9, 33, 38, 39]. This further confirms that the association between screen and central obesity is independent of confounders. It should also be noted that the included studies were performed between 2012 and 2020; in this period, the prevalence of screen use has dramatically increased among children and adolescents. According to the report entitled “Children and parents: media use and attitudes report 2019”, the screen use and ownership among children aged 5–15 years increased from 35% in 2015 to 45% in 2019, which is mostly attributed to tablet and mobile cellphone use [48]. At the same time, the increased prevalence of central obesity occurred among children and adolescents [49, 50]; therefore, studying the association between central obesity and screen time could not be affected by time.

Due to the relatively limited number of included studies, the results of subgrouping might not be representative. However, community-based studies are more reliable than school-based studies because they show a greater relationship between central obesity and screen time.

We sub-grouped our results according to the type of screen. In our meta-analysis, by removing the results of video games reported in the study by Castro JAC et al*.* [9], there was a significant change in the results. In the literature review, we witnessed this inconsistency in the health effects of video games in different studies. While one study reported higher WC among those playing video games for more than 2 h per day, in a meta-analysis by Gao Z et al. [51], playing active video games was associated with beneficial health effects like increased heart rate, energy expenditure, and oxygen consumption. However, the effect size was only meaningful when playing active video games was compared with sedentary behaviors and not with regular physical activities. Therefore, they could only be suggested as a good alternative to sedentary behavior. Further studies are needed to better elucidate the association between video games and health among children and adolescents [52].

In the current study, odds of central obesity was not associated with screen time that might be attributed to the high heterogeneity values of the included studies (62%). In sensitivity analysis, using the leave-one-out method, by removing the video game result of the study by Castro JAC et al*.* [9], the results were significant (OR 1.196; 95% CI 1.024–1.397; *P* = 0.024). This further clears that the diverse screen tools might show different results and further studies in different screen tools with help to better clarify the screen time and central obesity association. We observed higher WC in those with the highest versus lowest screen time. High screen time, as a sedentary behavior, could be associated with increased obesity risk via increased fat deposition in vessels or adipose tissue, particularly the visceral abdominal area [53–55]. Second, increased screen activities are associated with increased food intake. Numerous studies confirmed that watching television increases motivated response to food intake and snacking behavior among children and adolescents [56–59]; this is also true for video games [60–62] and PC use [63, 64]. Therefore, the association between screen time and increased central or general obesity is bidirectional. Due to the cross-sectional design of the included studies in this meta-analysis, reverse or reciprocal causality is possible, where adiposity or obesity may lead to increased screen time. More importantly, food-related advertisements on TV can potentially affect children’s food behaviors by promoting junk food and fast food consumption, and increase obesity risk [65–70]. Therefore, the association between central obesity and screen use is a multi-dimensional problem and all its aspects need to be studied.

## Conclusion

For the first time, this meta-analysis study revealed higher WC in children and adolescents with highest screen time versus lowest screen time. Although, odds of central obesity was not associated with screen time in this age group. This might be attributed to high heterogeneity of the included studies and the diversity of screen tools that were used in different studies. Therefore, further studies with interventional or observational design will help to evaluate this association more logically. Also, due to the observational design of the included studies, by recruiting further interventional or longitudinal studies, the causality inference will be possible in the future.

## Supplementary Information

**Additional file 1:** **Table S1.** PRISMA Checklist. **Table S2.** Search strategies and the number of records according to different electronic database. **Figure S1.** Begg’s funnel plot (with pseudo 95% CIs) of the odds ratios of the association between screen time and central obesity among children and adolescents (A); the comparison of waist circumference (WC) between highest versus lowest screen time categories (B).
